# Sex-specific relations of ceramides and white matter hyperintensities in the Rhineland study

**DOI:** 10.1038/s41598-025-06911-z

**Published:** 2025-07-03

**Authors:** Elvire N. Landstra, Valerie Lohner, Monique M.B. Breteler

**Affiliations:** 1 Population Health Sciences, German Centre for Neurodegenerative Diseases (DZNE), Venusberg-Campus 1, Building 99, 53127 Bonn, Germany; 2 Cardiovascular Epidemiology of Aging, Department of Cardiology, Faculty of Medicine and University Hospital Cologne, University of Cologne, Cologne, Germany; 3 Institute for Medical Biometry, Informatics and Epidemiology (IMBIE), Faculty of Medicine, University of Bonn, Bonn, Germany

**Keywords:** Ceramides, Brain lipids, Epidemiology, White matter hyperintensities, Sex differences, Menopause, Epidemiology, Sphingolipids, Neurovascular disorders

## Abstract

Women have a higher white matter hyperintensity (WMH) burden compared to men of a similar age, but the causes of this remain unclear. Ceramide (CER) levels differ between men and women and were previously linked to WMH, suggesting they could underlie these sex differences. Therefore, we associated twelve CERs, CER totals, six CER ratios, and a CER score reflecting the CERs most strongly associated with cardiovascular disease, with WMH load in 3,283 participants (30–95 years) of the community-based Rhineland Study. We assessed sex-specific relations between CERs and WMH by including an interaction term and performing sex-stratified analyses. We further investigated the influence of menopause by comparing relations in pre- and postmenopausal women of the same age. Multiple CERs, CER ratios, and the CER score were associated with increased WMH load, with most associations being significantly stronger in women than in men. We found no influence of menopausal status on the associations between CERs and WMH. Thus, we showed novel and strong associations of CERs, CER ratios and the CER score with WMH burden, especially in women. This study suggests that CERs may play an important role in the observed sex differences in WMH burden.

**Supplementary Information:**

The online version contains supplementary material available at 10.1038/s41598-025-06911-z.

## Introduction

Women, particularly after menopause, have a higher burden of white matter hyperintensities of presumed vascular origin (WMH) than men^1^. WMH are a prominent marker of cerebral small vessel disease (cSVD)^2^, have been linked to cardiovascular risk factors^3,4^, and are associated with elevated risk of developing stroke^5^, motor impairments^6,7^, mood disorders^8,9^, and cognitive dysfunction^5,10,11^.

Ceramides (CERs) are important structural and signaling lipids that are crucial for maintaining homeostasis and may have an impact on various pathogenetic processes^12^. Although more than 200 distinct CER species have been identified in mammals and across different tissues^13^, human studies usually include only a smaller subset of the more abundant ones. Variation in the degree of saturation and length of a CER’s fatty acid (FA) tail is regulated by different ceramide synthases (CerS)^14^, and creates a diverse range of CER species. The relation between CER species and health outcomes depends on this FA tail. Higher levels of some of these species, particularly CER16:0, CER18:0 and CER24:1, have been linked to cellular dysfunction^15,16^, dementia^17–20^, cancer^18^, atherosclerosis^21^, cardiovascular disease (CVD)^15,18,22,23^, and type 2 diabetes (DM2)^15,16,18,23^. In contrast, persons with higher concentrations of CER22:0 and CER24:0, in particular relative to other CERs, reportedly have a lower risk of dementia, suggesting that these CERs could be beneficial^15,19^. Not only have CERs been related to various diseases, but studies also support a possible causal link. Major prospective studies and animal studies confirmed that increased CER concentrations preceded and predicted disease, and showed a dose-reponse effect^22,24–27^. Several mechanisms have been suggested, including CERs causing endothelial dysfunction, inducing inflammation, disrupting the insulin-signaling cascade, and promoting LDL aggregation in the vessel wall^24,28–31^. Of note, previous studies have found sex differences in CER concentrations^12,32,33^. In particular, CER levels were generally lower in younger, but higher in older women compared to men, suggesting that these sex differences are influenced by hormones^12,33,34^. While menopause has been suspected to influence CERs, this has not been investigated to the best of our knowledge.

It has been suggested that CERs may play a role in WMH pathogenesis^12,32,35^. In previous population studies of older adults, higher concentrations of CER16:0, as well as the ratios of CER16:0/24:0 and CER24:1/24:0 were found to be associated with a higher WMH burden^19,32^, whereas CER24:0 and the ratio of CER22:0/16:0, were associated with a lower burden^19^. Additionally, a CER score, reflecting the CERs most strongly associated with CVD^25^, was also associated with WMH^32^.

Despite the clinical relevance of WMH and the potential role of CERs in its pathogenesis, sex-specific associations between CERs and WMH across the adult lifespan remain unknown. Moreover, while previous studies have investigated the effect of certain CERs and CER ratios (CER16:0/24:0, CER18:0/24:0, CER24:1/24:0, CER22:0/16:0, CER24:0/16:0), others (CER18:0/16:0, CER20:0/24:0) have not yet been assessed in relation to WMH^19,32^. In the present study, we therefore quantified the relation of multiple CERs and CER ratios with WMH load in a community-based population aged 30–95 years. Specifically, we investigated sex- and menopause-specific relations between CERs and WMH burden.

## Results

### Study population

Our study population had a mean age of 54.3 (SD: 13.7) years and 57% were women (*n* = 1,881) (Table 1). Of the women, 1,083 (57%) were postmenopausal. Women had a higher WMH load and lower BMI compared to men independent of age, while more men used lipid-lowering medication.

**Table 1:** Characteristics of the total study population, stratified by sex. WMH load is displayed as median [Interquartile range (IQR)]. Age is displayed as mean (range, standard deviation (SD)). All other values are presented as mean (SD) for continuous variables, or number of participants (percentages) for categorical variables. WMH: White matter hyperintensity, BMI: Body mass index, HDL-C: high-density lipoprotein cholesterol, LDL-C: low-density lipoprotein cholesterol, CVD: history of cardiovascular disease, AAT: abdominal adipose tissue, VAT/SAT: ratio between visceral and subcutaneous adipose tissue. .

Characteristics	All participants (*N* = 3,283)	Women (*N* = 1,880)	Men (*N* = 1,403)	*p*-value**
Age (years)	54.3 (30–95; 13.7)	54.6 (30–95; 13.5)	54.0 (30–88; 13.9)	0.283
Women	1,880 (57)			
Education				
* Low*	78 (2)	63 (3)	15 (1)	**< 0.001**
* Middle*	1,378 (42)	904 (48)	474 (34)	
* High*	1,827 (56)	913 (49)	914 (65)	
Menopause	1083 (33.0)	1083 (57.6)		
WMH load (%)	0.11 [0.05, 0.27]	0.12 [0.05, 0.30]	0.10 [0.05, 0.25]	**0.001**
BMI (kg/m^2^)*	25.56 (4.14)	25.18 (4.59)	26.06 (3.39)	**< 0.001**
Smoking*	426 (13)	228 (12)	198 (14)	0.110
Use of lipid-lowering medication*	359 (18)	166 (15)	193 (22)	**< 0.001**
Diabetes*	145 (4)	61 (3)	84 (6)	**< 0.001**
Hypertension*	1,158 (35)	607 (32)	551 (39)	**< 0.001**
History of CVD*	547 (17)	314 (17)	233 (12)	0.926
HDL-C*	63.23 (17.95)	70.30 (17.19)	53.71 (14.15)	**< 0.001**
LDL-C*	127.08 (35.71)	126.50 (36.87)	127.87 (34.08)	0.197
Triglycerides*	110.27 (69.97)	96.66 (48.36)	128.60 (88.09)	**< 0.001**
VAT/SAT*	0.513 (0.375)	0.308 (0.171)	0.785 (0.400)	**< 0.001**
AAT (liters)*	4.68 (2.35)	4.40 (2.39)	5.05 (2.24)	**< 0.001**

^*^Participants with missing data: BMI (n=11); Smoking (n=1); Use of lipid-lowering medication (n=38); Diabetes (n=24); Hypertension (n=34); CVD (n=5); HDL-C (n=104); LDL-C (n=104); Triglycerides (n=104); CVD (n=5); AAT (n=80); VAT/SAT (n=80).

^**^P-values comparing women and men, adjusted for age where applicable.

The absolute and relative concentrations of most CERs substantially differed between women and men (Supplementary Table 1). Although absolute concentrations of CER14:0, CER18:1, CER22:1, CER26:0 and CER26:1 were higher in women compared to men, the total concentrations of CER as well as CER22:0, CER24:0, and CER24:1 were higher in men. Relative concentrations of %CER14:0, %CER16:0, %CER18:0, %CER18:1, %CER20:0, %CER20:1, %CER22:1, %CER26:0, %CER26:1 were higher in women compared to men, whereas men had higher relative concentrations of total %CER, %CER22:0, and %CER24:0.

### Correlations between cers, WMH load, demographics, and cardiovascular risk factors

**Fig. 1:**
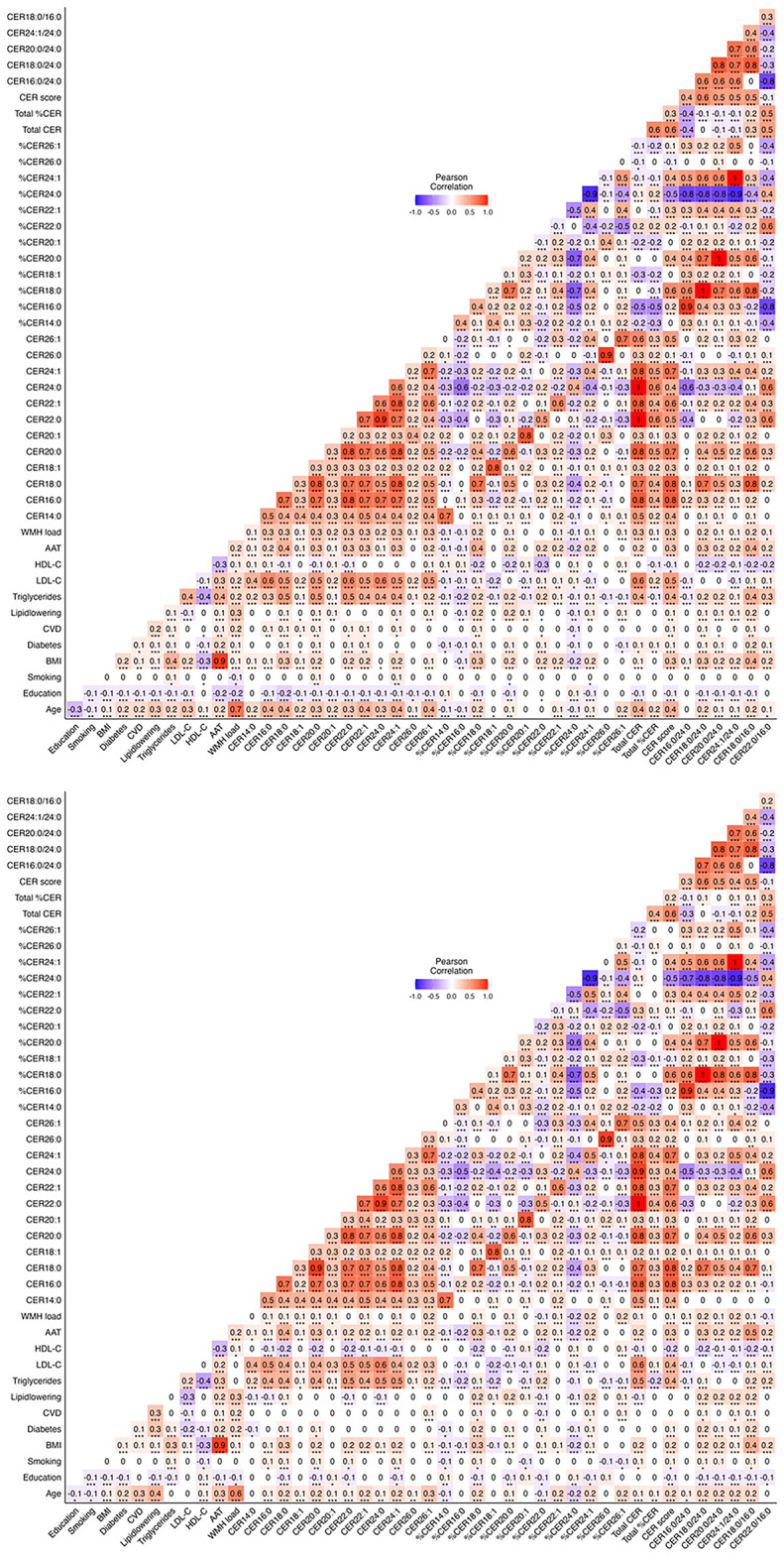
Correlations between concentrations of CERs, WMH load, demographics, and cardiovascular risk factors for women (**A**) and men (**B**) separately. The strength and direction of the correlation is represented by the colour and number, while significance is represented with * (*p* < 0.05), ** (*p* < 0.01), or *** (*p* < 0.001). BMI: body mass index; CER: ceramide; CVD: cardiovascular disease; HDL-C; high-density lipoprotein cholesterol; LDL-C: low density lipoprotein cholesterol; WMH: white matter hyperintensity.

Correlograms showing correlations between all CERs, WMH load, demographics, and cardiovascular risk factors in the data, are shown in Fig. 1 for men and women separately. Of note, age was more strongly correlated with higher levels of almost all CERs in women compared to men. In women, WMH load was correlated with higher levels of all CERs except %CER18:1, %CER20:1, %CER22:0, %CER26:0, and CER16:0/24:0. In men, the correlations were fewer and less strong.

### CERs are related to a higher WMH burden

**Fig. 2:**
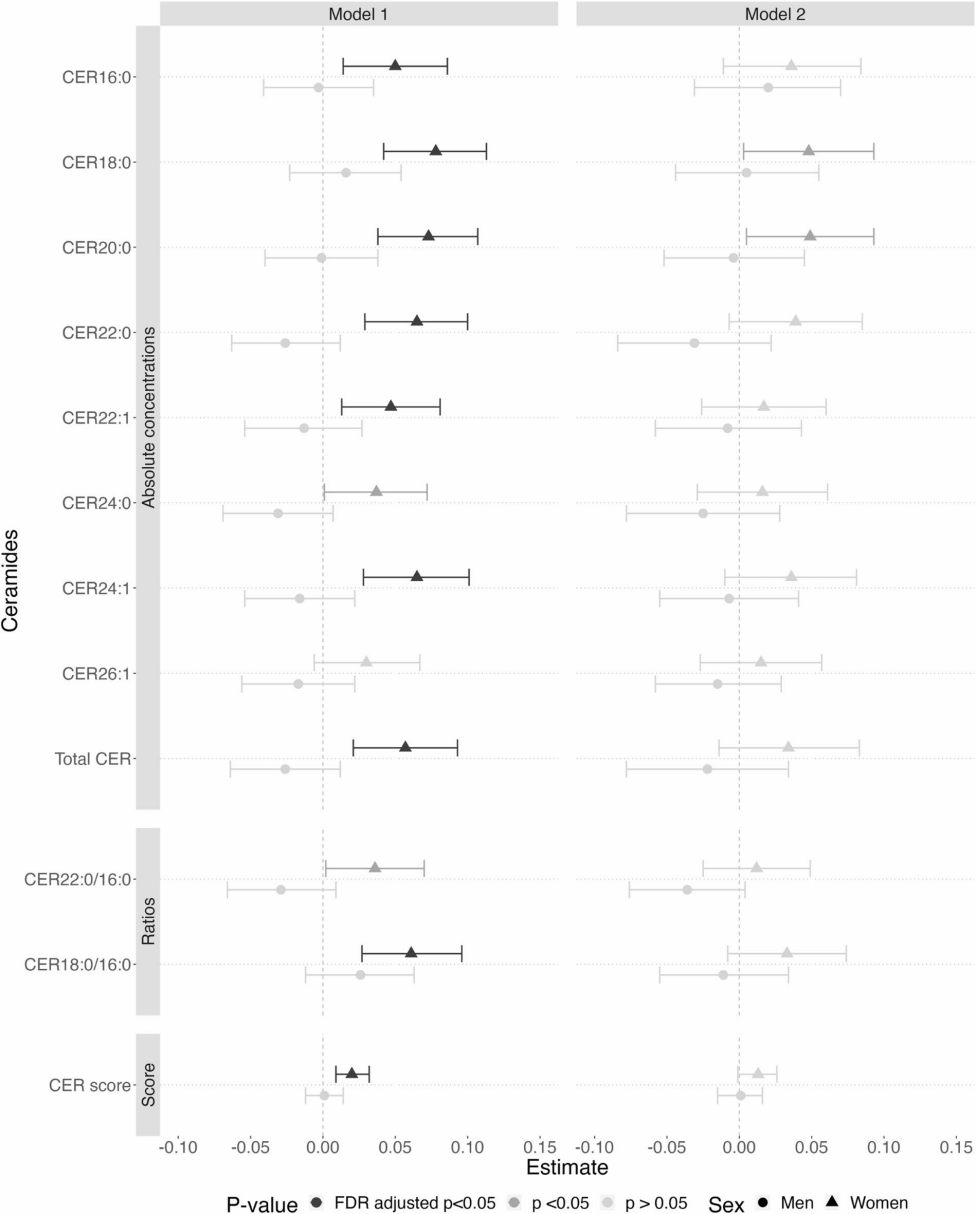
Beta-estimates with 95% confidence interval for associations between CER measures and WMH load. Plotted beta-estimates with 95% confidence intervals (x-axis) for the associations between the absolute and relative CER concentrations, CER ratios, and the CER score, and WMH load. Model 1 was adjusted for age, age-squared, and sex. Model 2 was additionally adjusted for education, abdominal adipose tissue, smoking, use of lipid-lowering medication, as well as levels of high-density lipoprotein cholesterol, low-density lipoprotein cholesterol, and triglycerides. Colour signifies significance, with light grey meaning non-significant, grey being nominally (*p* < 0.05) significant and dark grey being significant at an FDR-adjusted threshold (FDR-corrected *p* < 0.05). P values of the sex interaction term from an additional model are shown per CER. CER: ceramide; WMH: white matter hyperintensity.

Figure 2 shows that absolute levels of CER18:0 and CER20:0 were significantly associated with a higher WMH load independent of sex, age and age-squared (CER18:0 beta estimate per increase in one SD (ß) = 0.056 [95% confidence interval (CI) 0.030, 0.082], and CER20:0 ß = 0.046 [95%CI 0.020, 0.072]). Higher concentrations of CER16:0, CER22:0, CER22:1, and CER24:1 were associated with a higher WMH load at a nominal, but not FDR-corrected, significance level (CER16:0 ß = 0.032 [95%CI 0.006, 0.058], CER22:0 ß = 0.030 [95%CI 0.004, 0.056], CER22:1 ß = 0.027 [95%CI 0.001, 0.053], and CER24:1 ß = 0.030 [95%CI 0.004, 0.057]). The effect sizes of these associations attenuated slightly and became largely non-significant upon additional correction for education, smoking, use of lipid-lowering medication, LDL-C, HDL-C, triglycerides, and AAT.

Relative CER concentrations largely showed the same pattern: When taking into account sex, age, and age-squared, higher levels of %CER18:0 were associated with a higher WMH load after FDR adjustment, while higher levels of %CER20:0 were associated with a higher WMH burden at a nominal significance level (%CER18:0 (ß = 0.044 [95%CI 0.019, 0.07], and %CER20:0 ß = 0.033 [95%CI 0.008, 0.058]) (Fig. 2). Further adjustment for education, smoking, use of lipid-lowering medication, LDL-C, HDL-C, triglycerides, and AAT attenuated all associations.

Higher ratios of CER18:0/16:0, CER18:0/24:0, and CER20:0/24:0 were associated with more WMH independent of sex, age and age-squared (CER18:0/16:0 ß [95% CI] = 0.047 [0.022, 0.073], CER18:0/24:0 ß [95% CI] = 0.043 [0.017, 0.068], CER20:0/24:0 ß [95% CI] = 0.036 [0.010, 0.061]) (Fig. 2). However, effect sizes decreased and became non-significant upon further adjustment (Fig. 2). The previously associated ratios of CER16:0/24:0, CER22:0/16:0, CER24:1/24:0, and CER22:0/16:0 were not associated in either model (Model 1: CER16:0/24:0 ß [95% CI] = 0.010 [−0.015, 0.036], CER22:0/16:0 ß [95% CI] = 0.010 [−0.015, 0.035], CER24:1/24:0 ß [95% CI] = 0.016 [−0.009, 0.042]; Model 2: CER16:0/24:0 ß [95% CI] = 0.009 [−0.017, 0.036], CER22:0/16:0 ß [95% CI] = −0.007 [−0.034, 0.020], CER24:1/24:0 ß [95% CI] = 0.006 [−0.021, 0.033]). Lastly, the CER score was associated with a higher WMH burden upon adjustment for sex, age and age-squared, but not after additional adjustment for education, smoking, use of lipid-lowering medication, LDL-C, HDL-C, triglycerides, and AAT (age- and sex adjusted: CER score ß = 0.013 [95%CI 0.005, 0.022]; additional adjustment for cardiovascular risk factors: CER score ß = 0.008 [95%CI −0.002, 0.018]) (Fig. 2). Sensitivity analyses excluding people with possible or probable dementia status (*n* = 5) did not substantially change these results (Supplementary Table 2).

Next, we tested our hypothesis of cardiometabolic disease indicators being on the causal pathway by performing a mediation analysis with CVD, hypertension and diabetes as mediators (Supplementary Table 3). In particular, we found that 17.8% and 19.3% of the association between CER18:0 and CER24:1, respectively, and WMH was mediated through hypertension. Neither diabetes nor CVD were found to mediate more than 5% of the associations between absolute CER concentrations and WMH. For relative concentrations, between 20% and 29% of the associations between %CER14:0, %CER18:0, %CER22:0, %CER24:0, and %CER24:1, and WMH was mediated through hypertension. Diabetes mediated 15.4% and 12.5% of the association between %CER22:0 and %CER26:1 and WMH. CVD mediated less than 1% of the associations between relative CER concentrations and WMH. Similarly, the effect of the ratios and CER score was largely mediated through hypertension (max 42.6% and 16.7%, respectively).

### CERs have a stronger effect on WMH load in women compared to men

**Fig. 3:**
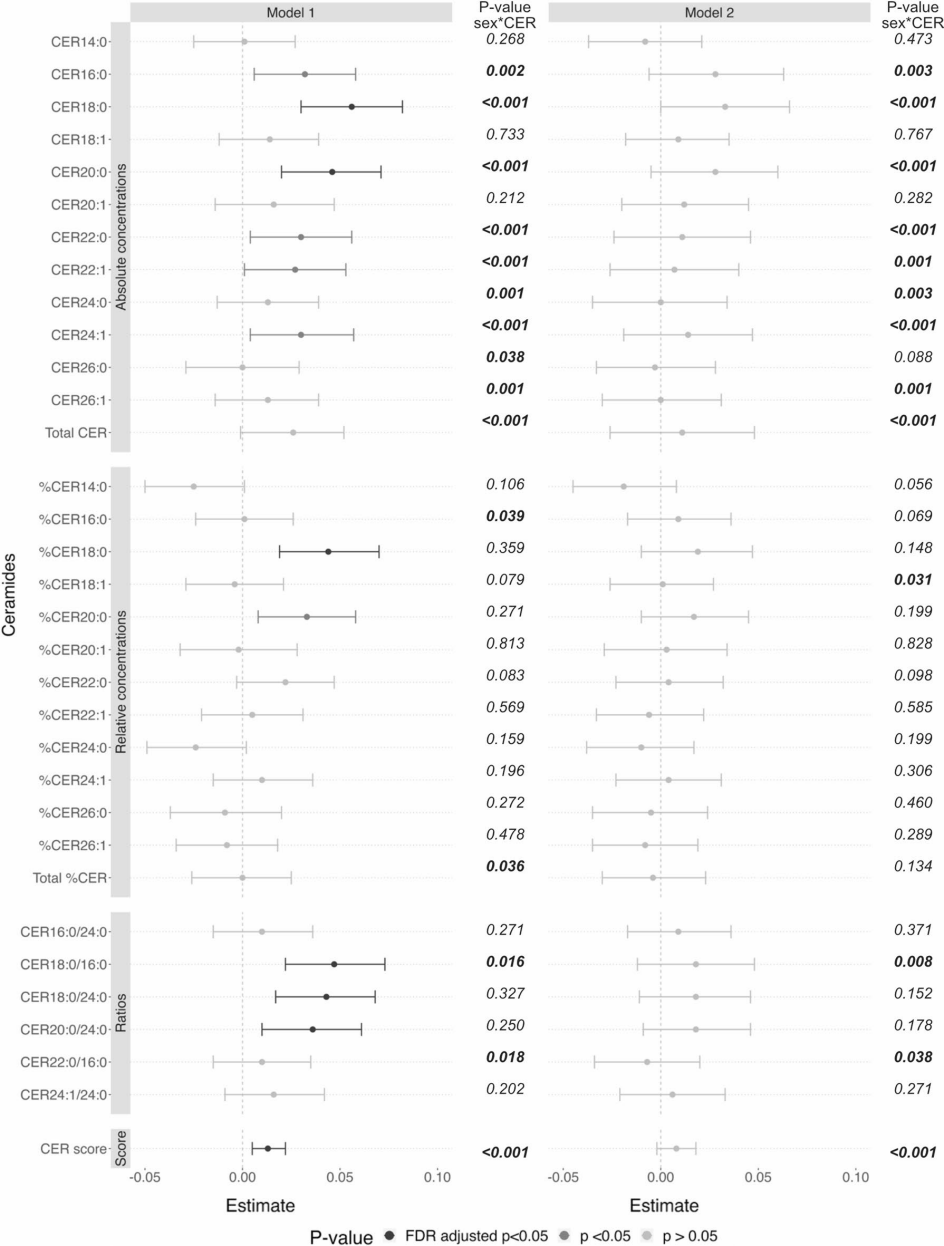
Sex-stratified estimates for associations between CER measures and WMH load. Sex-stratified beta estimates for absolute and relative CER concentrations, CER ratios and the CER score with a significant CER*sex interaction in the overall sample are displayed with 95% confidence intervals. Model 1 was adjusted for age and age-squared. Model 2 was additionally adjusted for education, abdominal adipose tissue, smoking, use of lipid-lowering medication, as well as levels of high-density lipoprotein cholesterol, low-density lipoprotein cholesterol, and triglycerides. Colour signifies significance, with light grey meaning non-significant, grey being nominally (*p* < 0.05) significant and dark grey being significant at an FDR-adjusted threshold (FDR-corrected *p* < 0.05). Shape indicates the sex, with triangles depicting women and circles depicting men. CER: ceramide; WMH: white matter hyperintensity.

We found significant sex interactions for most absolute concentrations of CERs, three ratios and the CER score. Upon subsequent sex-stratified analyses, we found that higher absolute concentrations of CER16:0, CER18:0, CER20:0, CER22:0, CER22:1, CER24:1, the ratio of CER18:0/16:0, as well as the CER score were associated with a significantly higher WMH load in women, but not in men (Fig. 3). For CER24:0 and CER22:0/16:0, we saw the same pattern, but associations were only nominally significant. Upon additional adjustment for AAT, smoking, LDL-C, HDL-C, triglycerides, and use of lipid-lowering medication most associations attenuated and were no longer statistically significant, with the exception of CER18:0 and CER20:0 which remained associated with WMH in women at a nominal significance level. Nonetheless, the difference in the magnitude of the sex-specific estimates remained. Results were also similar after the exclusion of people with possible or probable dementia status (Supplementary Table 2).

### Influence of menopause on the association between CERs and WMH load

Next, we assessed the effect of menopause on the association between CERs and WMH load in pre- and postmenopausal women in the overlapping age range (45–57 years). Of the 602 women, 310 (51.5%) were postmenopausal. As could be expected, the average age of the postmenopausal women was higher than that of the premenopausal women in this overlapping age band (mean age (SD) postmenopausal women: 54.6 (2.6) years; premenopausal women: 48.9 (3.0) years; *P* < 0.001, Table 2). After adjustment for age, the postmenopausal women had a higher WMH load (median [interquartile range (IQR)] WMH load: pre-menopausal women: 0.07 [0.04, 0.13], post-menopausal women: 0.11 [0.06, 0.19], *P* = 0.008). Other characteristics were similarly distributed in pre- and postmenopausal women (Table 2). Age-adjusted differences in CER concentrations between pre- and postmenopausal women are shown in Supplementary Table 1. Post- compared to premenopausal women had significantly higher levels of total CER (*P* = 0.002) and %CER (*P* = 0.007), as well as higher levels of CER16:0 (*P* = 0.012), CER18:0 (*P* = 0.002), CER20:0 (*P* = 0.003), CER22:0 (*P* < 0.001), CER22:1 (*P* = 0.016), CER24:1 (*P* = 0.010), CER26:1 (*P* = 0.001), %CER22:0 (*P* = 0.036), CER18:0/16:0 (*P* = 0.024), CER22:0/16:0 (*P* = 0.027), and a higher CER score (*P* = 0.006), but lower levels of CER24:0 (*P* = 0.008) and %CER18:1 (*P* = 0.046).

**Table 2:** Characteristics of women of menopausal age, stratified by menopausal status. Ceramide concentrations in the total study population, stratified by sex, and in women of menopausal age, stratified by menopausal status. All values are presented as mean (SD). CER: ceramide.

Characteristics	Premenopausal (*N* = 292)	Postmenopausal (*N* = 310)	*p*-value**
Age (years)	48.9 (45–57; 3.0)	53.6 (45–57; 2.6)	**< 0.001**
Education			
* Low*	4 (1)	4 (1)	0.103
* Middle*	126 (43)	157 (51)	
* High*	162 (55)	149 (48)	
WMH load (%)	0.07 [0.04; 0.13]	0.11 [0.06; 0.19]	**0.008**
BMI (kg/m^2^)*	24.76 (4.84)	25.21 (4.50)	0.632
Smoking*	34 (11.6)	47 (15.2)	0.110
Use of lipid-lowering medication*	4 (1.4)	11 (3.5)	0.731
Diabetes*	3 (1)	8 (3)	0.958
Hypertension*	55 (19)	79 (18)	0.649
History of CVD*	37 (13)	41 (13)	0.838
HDL-C*	68.99 (15.95)	71.78 (17.99)	0.155
LDL-C*	121.43 (31.96)	130.77 (33.53)	0.271
Triglycerides*	88.44 (45.54)	97.32 (45.96)	0.266
VAT/SAT*	0.24 (0.12)	0.31 (0.13)	0.036
AAT (liters)*	3.87 (2.37)	4.51 (2.38)	0.243

^*^Participants with missing data: Smoking (n=1); Use of lipid-lowering medication (n=18); Diabetes (n=2); Hypertension (n=15); HDL-C (n=21); LDL-C (n=21); Triglycerides (n=21); AAT (n=6); VAT/SAT (n=6).

^**^P-values comparing pre-and postmenopausal women, adjusted for age where applicable.

Despite significant differences in the concentrations of CERs between pre- and postmenopausal women, we found no evidence for a different relation between CERs concentrations and WMH burden between pre- and postmenopausal women (all CER*menopause interaction terms *P* > 0.068 (median P value [IQR]: 0.677 [0.439, 0.824]).

## Discussion

In this population-based cohort study, we found that absolute concentrations of CER18:0, CER20:0, the relative concentration of %CER18:0, the ratios of CER18:0/16:0, CER18:0/24:0, CER20:0/24:0, and the CER score, were significantly associated with a higher WMH load. Moreover, several other CERs were associated with a higher WMH load before FDR adjustment (CER16:0, CER22:0, CER22:1, CER24:1, %CER20:0). Interestingly, we found a significant interaction between CERs and sex for eight out of 12 absolute CER concentrations, two out of six ratios, and the CER score, but none of the relative concentrations. Sex-stratified analyses showed that these CERs and CER measures were consistently associated with a higher WMH load in women but not in men. Although the concentrations of CERs differed between pre- and postmenopausal women of a similar age, and postmenopausal women had a higher WMH load than pre-menopausal women, we found no significant interaction between CERs and menopause on WMH burden. Overall, our results suggest that CERs might, to an extent, play a role in sex-specific mechanisms of WMH.

Our finding that CER16:0 and the CER score are associated with more WMH, aligns with previous work on WMH^19,32,36^. We extended these previous results with newly found associations between other CERs and WMH. Interestingly, most of the CERs we found to be associated with WMH had already been found to be related to CVD in previous research (CER18:0, CER20:0, CER24:1, CER22:0, CER18:0/24:0, CER20:0/24:0)^25,37–39^, and were even suggested as possible biomarkers^18,40^. Taken together, this suggests that CERs might also be candidate biomarkers for cSVD, and that these CERs might play a role in both CVD and WMH through a common mechanism. For example, higher levels of these CERs could stimulate inflammation and apoptosis^40^. Although CERs have been shown to precede myocardial infarction in rodents and predict CVD in humans^22,25,37,41^, longitudinal research is still necessary to determine temporality in their relation to WMH before it could be considered as a clinical marker or a possible cause. Nonetheless, investigating ways of reducing CERs in vivo, such as certain enzymatic reactions^42^, could be beneficial.

We found that CER16:0, CER18:0, CER20:0, CER22:0, CER22:1, CER24:0, CER24:1, CER18:0/16:0 and CER22:0/16:0 were strongly associated with a higher WMH burden in women but not in men, whereas we found significant relations with WMH in both sexes for %CER18:0, %CER20:0, CER18:0/16:0, CER18:0/24:0 and CER20:0/24:0. This is in contrast with a previous study in older people that reported no difference in the size of the effect estimates between men and women for the ratios and absolute concentrations of CERs^32^. However, that study only included 588 persons, and while we confirmed their finding that women had higher concentrations of certain CERs compared to men, they may have been underpowered to reveal existing sex-differences in the association with WMH. Little human research exists to explain what might underlie the sex-specific associations that we found. Most animal studies have focused on male animals only, but one previous study in mice studied sexual dimorphism and showed clear sex differences in the regulation of hepatic CERs, and their effects on insulin resistance^43^. Interestingly, although postmenopausal women had higher levels of almost all CERs, and a higher WMH burden, we found no evidence of effect modification by menopausal status. These higher CER concentrations after menopause might be due to a change in the composition of visceral and subcutaneous fat, which has been suggested to affect CERs synthesis^12,34^. Generally, men have more VAT compared to women, but VAT increases in women after menopause^43,44^. Our hypothesis that body composition could possibly explain menopause-specific differences in the relation between CERs and WMH may therefore sound contradictory at first. However, our findings might imply that there are different mechanisms underlying the WMH pathologies in men and (pre-and postmenopausal) women, and that in women body composition might explain some of the differences in CER levels. Future studies are needed to further investigate the relationship between body fat composition and CER metabolism and possible sex-differences therein.

We presented models with and without adjustment for education, body fat composition, smoking, HDL-C, LDL-C, triglycerides, and use of lipid-lowering medication. Inclusion of these factors was based on previous literature suggesting they might be related to both the exposure and the outcome. However, the relations between these factors in the body is unlikely to be this simple. There are likely multiple feedback loops. Therefore, it is a matter of debate whether these cardiovascular risk factors should be adjusted for. As a higher body fat composition, smoking, and use of lipid-lowering medication may lead to an overabundance of saturated fatty acids as well as elevated cytokine levels, they can lead to elevated CER levels in the blood^38,45,46^. Correlations between these factors and other vascular damage, which may lead to de novo CER synthesis^27^, could further muddle the relations. To the extent that they are indeed causally related to CER concentrations, including these cardiovascular risk factors in the models would lead to overadjustment and possible underestimation of the strength of the true relationship between CERs and WMH. We observed that adjustment for these factors, indeed attenuated all associations between CERs and WMH load. In men, adjustment for these cardiovascular risk factors did not change the estimates substantially, which is not-surprising given the lack of assocations between sex-specific CERs and WMH before adjustment. As shown in our correlation analyses, WMH load was also less strongly correlated with CERs in men than women, and more strongly with, for example, diabetes, suggesting that other factors are likely involved in men. Further supporting the interplay between cardiovascular risk factors and CERs, we showed that part of the relation between CERs and WMH was mediated through cardiometabolic disease indicators, especially through hypertension. Further work is needed to understand the interplay of CERs, cardiovascular risk factors, cardiometabolic disease, and WMH.

Our study has several strengths. Firstly, our large sample size, and the wide age range of our participants, allowed us to delve deeper into sex differences. Furthermore, our broad lipid panel allowed us to evaluate more CERs and relative concentrations of CERs in relation to WMH than previous studies. A limitation is that our analysis was based on cross-sectional data, so we cannot establish the order of events. One might hypothesize that the cardiovascular pathology underlying WMH could trigger an inflammatory response, which in turn could lead to increased CER production^40,47^. However, given existing data from animal studies and the evidence regarding large vessel disease^15,27,40,41,48^, where elevated CER concentrations precede disease development, we consider reverse causality a highly unlikely explanation for our observations. Another limitation concerns the stability of CER concentrations in blood. As CERs were only measured once, our study was not able to consider interindividual fluctuations in CER blood levels, for example on account of the circadian rhythm^49^. However, by strict standardization of the blood withdrawal procedure and measuring CERs in fasting blood samples taken in the morning, we minimized the measurement noise that such fluctuations could have caused. Furthermore, data on menopausal status were self-reported and there might have been some misclassifications. Lastly, the influence of socioeconomic factors should be assessed in more detail. Our study is composed of mainly people from a high or middle class. As such, we could not properly evaluate whether the relationship differs across these levels.

In summary, we identified novel associations between CERs and WMH burden within a large community-based cohort covering the adult life span. Importantly, our data suggest that CERs might play a crucial role in sex-specific mechanisms of WMH, as they were strongly associated with a higher WMH burden in women specifically, independent of menopause status.

Our study evaluated the associations between CERs and WMH and identified interactions between sex and CERs in the associations with WMH burden. As cSVD disproportionally affects women, CERs might be an interesting candidate for explaining these sex differences in the cerebrovascular disease burden. In conclusion, while CERs have already shown to be an important player in the pathogenesis of CVD, our study contributes evidence that they might play a role in cSVD as well.

## Methods

### Study population and experimental procedures

This cross-sectional study used data from the Rhineland Study, a community-based cohort study among inhabitants aged 30 years and above in two geographically defined areas in Bonn, Germany. Participation is possible by invitation only and the sole exclusion criteria is insufficient command of the German language to provide informed consent. Approval to undertake the study was obtained from the ethics committee of the University of Bonn, Medical Faculty. The study was carried out in accordance with the recommendations of the International Council for Harmonisation Good Clinical Practice standards. We obtained written informed consent from all participants in accordance with the Declaration of Helsinki.

The source population for the present study consisted of the first 5,000 participants of the Rhineland Study who completed their baseline assessment. Of these, we excluded people who did not undergo magnetic resonance imaging (MRI) (*n* = 1,418), failed the MRI quality control (*n* = 84), had a stroke or multiple sclerosis (*n* = 69), or had no CER data available (*n* = 146), leaving 3,283 participants to be included in the analyses.

### Assessment of ceramides

Blood plasma samples were collected from participants and sent to Metabolon for analysis (Metabolon, USA). The concentrations of twelve CER species as well as their total concentrations were extracted in the presence of internal standards using an automated butanol and methanol (BUME) extraction according to the method of Lofgren et al.^50^ Briefly, extracts were dried under nitrogen and reconstituted in ammonium acetate dichloromethane: methanol before transferal to vials for infusion-mass spectrometry analysis. Samples were run on a Shimadzu LC with nano PEEK tubing and the Sciex Selexlon-5500 QTRAP, which was operated in multiple reaction monitoring (MRM) mode with more than 1,100 MRMs. The absolute concentration (nmol) of CERs was obtained by taking the ratio of the signal intensity of the target CER with its corresponding internal standard and multiplying it by the concentration of its internal standard. Mol% concentrations were calculated relative to the total CER concentration. The total CER mol% concentration was calculated relative to the other lipid classes (*n* = 14) measured on the platform. Relative concentrations are indicated by % in front of the name, such as %CER18:0. Additionally, we computed the ratios of the absolute concentration of CER18:0, CER16:0, CER24:1 and CER20:0 over CER24:0 as well as CER18:0 and CER22:0 over CER16:0, because these have previously been associated with either cardiovascular risk factors, WMH, or both^19,23,32,37^. Lastly, a CER score was calculated which reflected the CERs most strongly associated with CVD according to the method by Laaksonen et al.^25^ Briefly, for the concentrations of CER18:0, CER16:0, CER24:1 and their ratios with CER24:0, participants were given a score of 2 or 1 for being in the highest or second highest quartile respectively, resulting in a total score with a range between 0 and 12.

### MRI data acquisition and image analysis

All eligible participants underwent 3 Tesla MRI on a Siemens Magnetom Prisma (Erlangen, Germany) equipped with an 80 mT/m gradient system and a 64-channel phased-array head-neck coil. The MRI protocol and assessment of WMH and abdominal tissue in the Rhineland Study have been described in detail elsewhere^51^. In brief, WMH were defined as hyperintense signals in the white matter tracts on T2-weighted images^2^. WMH were quantified using an in-house developed pipeline, based on image information from the T1-weighted, T2-weighted, and FLuid-Attenuated Inversion Recovery (FLAIR) images. White matter volume was extracted using FreeSurfer’s automated segmentation atlas (aseg)^52^. Measurements of adipose tissue (abdominal adipose tissue (AAT), subcutaneous adipose tissue (SAT), and visceral adipose tissue (VAT)) were automatically derived from abdominal 3D Dixon images using the FatSagNet segmentation tool. To account for size differences between people the proportion of AAT volume relative to the height of the segmented area was calculated.

### Covariates

Covariates included sex (women/men), age, education (low/middle/high), AAT, current smoking status (yes/no), regular use of lipid-lowering medication (yes/no), high-density lipoprotein cholesterol (HDL-C), low-density lipoprotein cholesterol (LDL-C), triglyceride levels, hypertension (yes/no), diabetes (yes/no), and history of CVD (yes/no). Education was defined as the highest educational attainment and categorised according to the International Standard Classification of Education (ISCED) 2011 into low (completed lower secondary education or below), middle (completed upper secondary education up to completed Bachelor’s degree or equivalent), or high (completed Master’s degree or equivalent up to completed doctoral degree or equivalent). Smoking status was self-reported and missing values (*n* = 102) were imputed based on cotinine levels in the blood, as measured on the Metabolon HD4 platform, where the 97.5th percentile of cotinine levels in non-smokers was set as cut-off^53^. HDL-C, LDL-C and triglyceride levels were determined clinically. Menopausal status (premenopausal/postmenopausal) was self-reported. Women were considered postmenopausal when they were above the age of 60 years, had undergone a bilateral ovariectomy, or had had no menstruation for more than a year not due to pregnancy, breastfeeding or contraceptives. Women below 60 years who had had a hysterectomy were excluded from the menopause-related analyses because their menopausal status could not be determined.

### Statistical data analysis

**Fig. 4:**
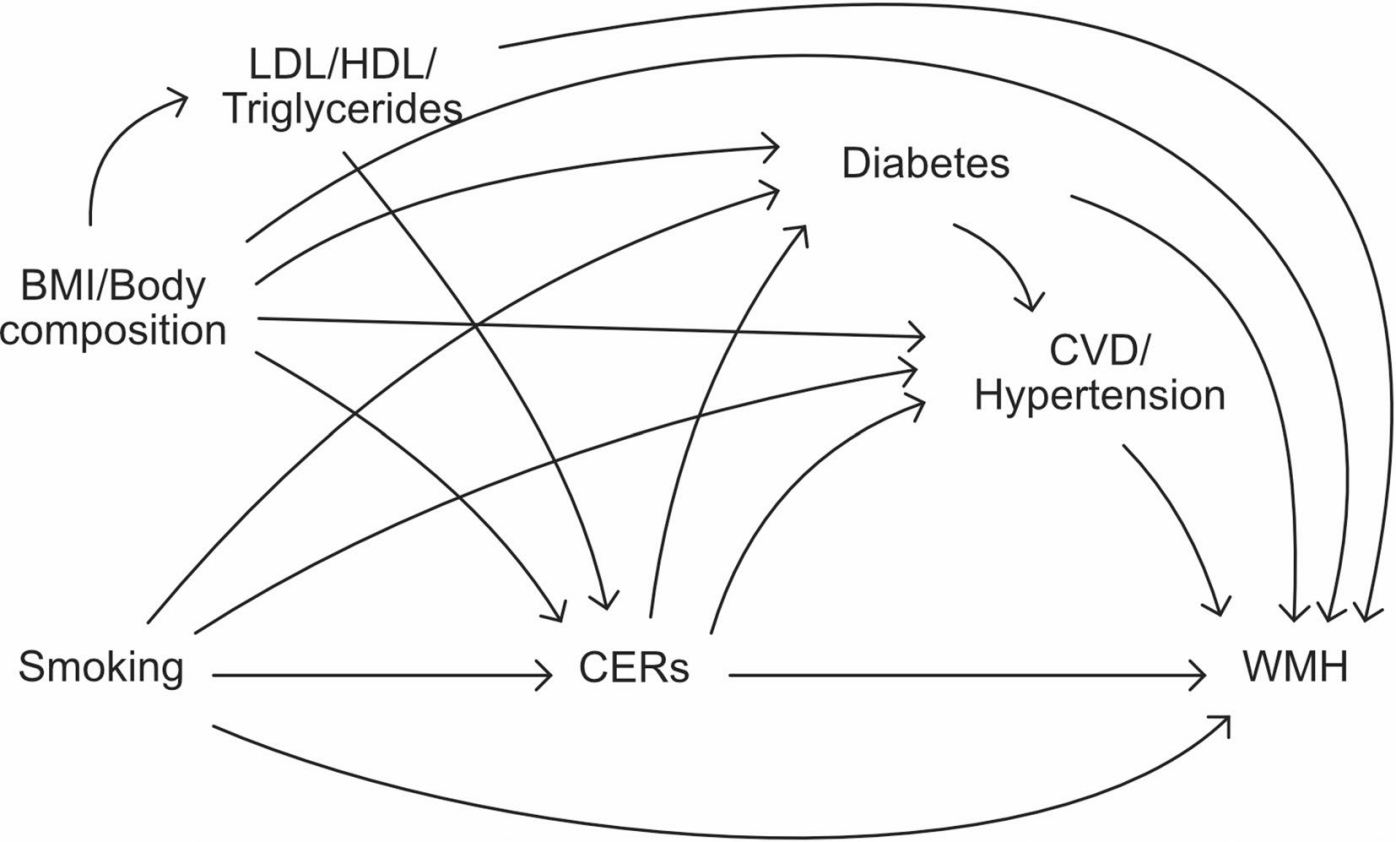
Directed acyclic graph showing associations between cardiovascular risk factors, cardiometabolic disease indicators, CERs and WMH. BMI: body mass index; CER: ceramide; CVD: cardiovascular disease; HDL-C; high-density lipoprotein cholesterol; LDL-C: low density lipoprotein cholesterol; WMH: white matter hyperintensity.

To adjust for brain atrophy, WMH lesion load was computed by dividing WMH volume by white matter volume. Due to the highly skewed distribution of the WMH load, we performed a logit-transformation and z-standardized it for interpretability. To investigate the correlations between all factors, we first ran Pearson correlations between CERs, WMH load, and the covariates. Next, we quantified the associations of standardized CER concentrations (absolute and relative), CER ratios and the CER score with WMH load using multiple linear regression models, adjusting for sex, age and age-squared to model non-linear age dependencies in model 1. In a further model we additionally adjusted for cardiovascular risk factors (education, smoking, AAT, HDL-C, LDL-C, triglycerides, and use of lipid-lowering medication). It should be noted that cardiometabolic disease indicators, such as hypertension and diabetes, may be on the causal pathway between lipid species and cerebral small vessel disease^18,21,27,38,40,41,47,54–58^. Inclusion of these factors in our models could thus lead to overadjustment. To test this hypothesis, we additionally performed a mediation analysis to test whether the effect of CERs on WMH were through cardiometabolic disease indicators (CVD, diabetes, hypertension). An overview of the presumed direction and relations between WMH, cardiovascular risk factors and cardiometabolic disease indicators is included in a directed acyclic graph in Fig. 4. As WMH are related to neurodegeneration, we also assessed whether associations were independent of dementia by exclusing people with possible or probable dementia (*n* = 5) dementia status in a sensitivity analysis.

Next, we evaluated whether the associations between CER levels and WMH load differed between sexes by including a sex*CER interaction term in the various models, followed by a sex stratified analysis if the sex*CER interaction term was significant (*p* < 0.05).

Furthermore, we investigated menopause-specific effects of CERs on WMH. For this, we used the same models as before with menopause and a menopause*CER interaction term added, and only including pre- and postmenopausal women in the overlapping age range. Postmenopausal women younger than 45 years were considered to experience early menopause and were excluded from this analysis. The oldest premenopausal woman in our dataset was 57 years old. We thus compared pre- and postmenopausal women between the ages of 45 and 57 years (*n* = 602). If the menopause*CER interaction term was significant (*p* < 0.05), we additionally performed analyses stratified by menopause status.

We corrected for multiple comparisons using a false-discovery rate (FDR) correction (Benjamini & Hochberg approach)^59^ separately for the relative and absolute CER concentrations, CER ratios and CER score. The number of effective tests was 13 for the relative and absolute concentrations, six for the ratios and one for the score. We considered a corrected P-value < 0.05 statistically significant. We performed all analyses in R version 4.2.1^60^, using the package “mediation” for the mediation analyses^61^.

## Electronic supplementary material

Below is the link to the electronic supplementary material.

Supplementary Material 1

## Data Availability

The datasets generated and analysed during the current study are not publicly available due to data protection regulations but are available from the corresponding author on reasonable request.
