# Supplementary material for: Sex-specific relations of ceramides and white matter hyperintensities in the Rhineland study
Source: Sci Rep. 2025 Jul 3;15:23757. doi: 10.1038/s41598-025-06911-z (PMC12229298; doi:10.1038/s41598-025-06911-z)
Supplement: Supplementary file 1 — Supplementary Material 1 [file 41598_2025_6911_MOESM1_ESM.pdf]

Sex-specific relations of Ceramides and White Matter Hyperintensities in the Rhineland Study

Elvire N. Landstra, Valerie Lohner, and Monique M.B. Breteler

Supplementary Table 1: Ceramide concentrations in the total study population, stratified by sex, and in women of menopausal age, stratified by menopausal status

**CER**	**Total sample**				**Women aged 45-57 years**		
	**All participants (N= 3,283)**	**Women (N = 1,880)**	**Men (N = 1,403)**	**p-value***	**Premenopausal (N = 292)**	**Postmenopausal (N = 310)**	**p-value****
Absolute concentrations (nmol/ml)							
CER14:0	0.016 (0.006)	0.017 (0.006)	0.015 (0.005)	<0.001	0.017 (0.005)	0.018 (0.006)	0.184
CER16:0	0.339 (0.080)	0.340 (0.078)	0.338 (0.082)	0.566	0.324 (0.066)	0.349 (0.074)	0.012
CER18:0	0.114 (0.043)	0.114 (0.043)	0.114 (0.044)	0.538	0.100 (0.034)	0.116 (0.041)	0.002
CER18:1	0.011 (0.005)	0.011 (0.005)	0.010 (0.005)	0.002	0.011 (0.005)	0.011 (0.004)	0.506
CER20:0	0.114 (0.036)	0.114 (0.036)	0.113 (0.036)	0.835	0.104 (0.029)	0.115 (0.033)	0.003
CER20:1	0.006 (0.003)	0.006 (0.003)	0.006 (0.003)	0.026	0.006 (0.003)	0.006 (0.003)	0.103
CER22:0	0.802 (0.237)	0.789 (0.232)	0.819 (0.243)	<0.001	0.734 (0.196)	0.838 (0.214)	<0.001
CER22:1	0.034 (0.0111)	0.035 (0.0113)	0.033 (0.0107)	<0.001	0.032 (0.010)	0.036 (0.010)	0.016
CER24:0	2.705 (0.765)	2.663 (0.753)	2.761 (0.776)	<0.001	2.512 (0.642)	2.845 (0.690)	0.008
CER24:1	1.204 (0.353)	1.185 (0.341)	1.229 (0.368)	<0.001	1.100 (0.279)	1.210 (0.322)	0.010
CER26:0	0.044 (0.034)	0.046 (0.038)	0.041 (0.028)	0.001	0.041 (0.024)	0.047 (0.027)	0.271
CER26:1	0.029 (0.011)	0.030 (0.011)	0.029 (0.011)	0.005	0.027 (0.008)	0.031 (0.011)	0.001
Total CER	5.405 (1.395)	5.336 (1.372)	5.497 (1.421)	<0.001	4.997 (1.149)	5.607 (1.218)	0.002
Relative concentrations (mol%)							
%CER14:0	0.308 (0.102)	0.330 (0.103)	0.280 (0.092)	<0.001	0.337 (0.098)	0.324 (0.104)	0.364
%CER16:0	6.384 (1.001)	6.486 (0.995)	6.247 (0.992)	<0.001	6.573 (0.937)	6.312 (0.959)	0.192
%CER18:0	2.110 (0.567)	2.133 (0.564)	2.079 (0.569)	0.01	2.004 (0.503)	2.064 (0.559)	0.166
%CER18:1	0.206 (0.0941)	0.212 (0.094)	0.197 (0.093)	<0.001	0.223 (0.113)	0.192 (0.075)	0.046
%CER20:0	2.102 (0.401)	2.132 (0.392)	2.062 (0.410)	<0.001	2.076 (0.357)	2.059 (0.377)	0.449
%CER20:1	0.111 (0.057)	0.114 (0.060)	0.107 (0.053)	0.002	0.112 (0.060)	0.111 (0.048)	0.373
%CER22:0	14.751 (1.337)	14.704 (1.320)	14.813 (1.357)	0.025	14.623 (1.258)	14.868 (1.390)	0.035
%CER22:1	0.633 (0.133)	0.658 (0.132)	0.600 (0.126)	<0.001	0.647 (0.140)	0.649 (0.122)	0.673
%CER24:0	49.906 (4.269)	49.758 (4.191)	50.106 (4.365)	0.029	50.116 (3.981)	0.649 (0.122)	0.725

%CER24:1	22.353 (3.441)	22.293 (3.325)	22.434 (3.591)	0.18	22.118 (3.204)	21.636 (3.287)	0.749
%CER26:0	0.814 (0.6403)	0.857 (0.7400)	0.755 (0.4708)	<0.001	0.825 (0.493)	0.840 (0.513)	0.578
%CER26:1	0.550 (0.1714)	0.566 (0.1629)	0.530 (0.1803)	<0.001	0.547 (0.138)	0.549 (0.147)	0.258
Total %CER	0.079 (0.015)	0.077 (0.013)	0.080 (0.016)	<0.001	0.075 (0.013)	0.080 (0.013)	0.007
Ratios							
CER18:0/16:0	0.333 (0.085)	0.332 (0.083)	0.336 (0.087)	0.098	0.307 (0.072)	0.329 (0.082)	0.023
CER22:0/16:0	2.371 (0.450)	2.324 (0.435)	2.434 (0.461)	<0.001	2.274 (0.405)	2.413 (0.452)	0.027
CER16:0/24:0	0.130 (0.028)	0.132 (0.028)	0.127 (0.028)	<0.001	0.133 (0.026)	0.126 (0.026)	0.557
CER18:0/24:0	0.043 (0.015)	0.044 (0.015)	0.043 (0.015)	0.018	0.041 (0.013)	0.042 (0.013)	0.178
CER20:0/24:0	0.043 (0.011)	0.044 (0.011)	0.042 (0.011)	<0.001	0.042 (0.010)	0.041 (0.010)	0.413
CER24:1/24:0	0.457 (0.108)	0.456 (0.104)	0.457 (0.113)	0.721	0.449 (0.101)	0.435 (0.102)	0.839
Score							
CER score	4.4 (3.1)	4.5 (3.1)	4.4 (3)	0.401	3.5 (2.7)	4.4 (2.9)	0.006

Legend: All values are presented as mean (SD). CER: ceramide

* P-values comparing women and men, adjusted for age.

** P-values comparing pre- and postmenopausal women, adjusted for age.

Supplementary Table 2: Beta estimates for associations between CERs and WMH load after exclusion of people with a possible or plausible dementia diagnosis (n= 5). Stratified by sex if the interaction term was significant (p<0.05). Model 1 was adjusted for sex, age, and agesquared. Model 2 was additionally adjusted for education, smoking, use of lipid-lowering medication, LDL-C, HDL-C, triglycerides, and AAT

**Lipid**	**Estimate**	**P value**	**Adj. p**	**Sex*CER p value**	**Women**			**Men**			**Model**
					**Estimate**	**P value**	**Adj. p**	**Estimate**	**P value**	**Adj. p**	
CER14:0	0 [-0.03 - 0.03]	0.948	0.994	0.273							Model 1
CER16:0	0.03 [0.01 - 0.06]	0.017	0.073	0.002	0.05 [0.01 - 0.09]	0.008	0.017	0 [-0.04 - 0.04]	0.881	0.964	Model 1
CER18:0	0.06 [0.03 - 0.08]	<0.001	<0.001	<0.001	0.08 [0.04 - 0.11]	<0.001	<0.001	0.02 [-0.02 - 0.05]	0.417	0.662	Model 1
CER18:1	0.01 [-0.01 - 0.04]	0.273	0.435	0.706							Model 1
CER20:1	0.04 [0.02 - 0.07]	0.001	0.007	<0.001	0.07 [0.04 - 0.11]	<0.001	<0.001	0 [-0.04 - 0.04]	0.964	0.964	Model 1
CER20:1	0.02 [-0.01 - 0.05]	0.301	0.435	0.209							Model 1
CER22:0	0.03 [0 - 0.06]	0.025	0.073	<0.001	0.06 [0.03 - 0.1]	<0.001	<0.001	-0.03 [-0.06 - 0.01]	0.186	0.605	Model 1
CER22:1	0.03 [0 - 0.05]	0.051	0.11	0.001	0.04 [0.01 - 0.08]	0.009	0.017	-0.01 [-0.05 - 0.03]	0.525	0.683	Model 1
CER24:0	0.01 [-0.01 - 0.04]	0.341	0.443	0.002	0.04 [0 - 0.07]	0.051	0.083	-0.03 [-0.07 - 0.01]	0.114	0.605	Model 1
CER24:1	0.03 [0 - 0.06]	0.028	0.073	<0.001	0.06 [0.03 - 0.1]	0.001	0.003	-0.02 [-0.05 - 0.02]	0.401	0.662	Model 1
CER26:0	0 [-0.03 - 0.03]	0.994	0.994	0.038							Model 1
CER26:1	0.01 [-0.01 - 0.04]	0.379	0.448	0.001	0.03 [-0.01 - 0.07]	0.119	0.172	-0.02 [-0.06 - 0.02]	0.398	0.662	Model 1
Total CER	0.03 [0 - 0.05]	0.062	0.115	<0.001	0.06 [0.02 - 0.09]	0.003	0.007	-0.03 [-0.06 - 0.01]	0.185	0.605	Model 1
CER14:0	-0.01 [-0.04 - 0.02]	0.598	0.864	0.497							Model 2
CER16:0	0.03 [-0.01 - 0.06]	0.108	0.468	0.004	0.04 [-0.01 - 0.08]	0.138	0.359	0.02 [-0.03 - 0.07]	0.446	0.878	Model 2
CER18:0	0.03 [0 - 0.07]	0.053	0.468	<0.001	0.05 [0 - 0.09]	0.037	0.24	0 [-0.04 - 0.06]	0.838	0.878	Model 2
CER18:1	0.01 [-0.02 - 0.04]	0.489	0.864	0.794							Model 2
CER20:1	0.03 [0 - 0.06]	0.099	0.468	<0.001	0.05 [0 - 0.09]	0.03	0.24	0 [-0.05 - 0.04]	0.878	0.878	Model 2
CER20:1	0.01 [-0.02 - 0.04]	0.449	0.864	0.281							Model 2
CER22:0	0.01 [-0.02 - 0.05]	0.532	0.864	<0.001	0.04 [-0.01 - 0.09]	0.099	0.359	-0.03 [-0.08 - 0.02]	0.254	0.878	Model 2
CER22:1	0.01 [-0.03 - 0.04]	0.694	0.902	0.002	0.02 [-0.03 - 0.06]	0.458	0.623	-0.01 [-0.06 - 0.04]	0.763	0.878	Model 2
CER24:0	0 [-0.04 - 0.03]	0.982	0.992	0.003	0.02 [-0.03 - 0.06]	0.495	0.623	-0.03 [-0.08 - 0.03]	0.362	0.878	Model 2
CER24:1	0.01 [-0.02 - 0.05]	0.404	0.864	<0.001	0.04 [-0.01 - 0.08]	0.135	0.359	-0.01 [-0.06 - 0.04]	0.778	0.878	Model 2
CER26:0	0 [-0.03 - 0.03]	0.862	0.992	0.091							Model 2
CER26:1	0 [-0.03 - 0.03]	0.992	0.992	0.001	0.01 [-0.03 - 0.06]	0.513	0.623	-0.01 [-0.06 - 0.03]	0.503	0.878	Model 2
Total CER	0.01 [-0.03 - 0.05]	0.568	0.864	<0.001	0.03 [-0.01 - 0.08]	0.173	0.375	-0.02 [-0.08 - 0.03]	0.442	0.878	Model 2

%CER14:0	-0.02 [-0.05 - 0]	0.067	0.2	0.111							Model 1
%CER16:0	0 [-0.02 - 0.03]	0.911	0.956	0.041							Model 1
%CER18:0	0.04 [0.02 - 0.07]	0.001	0.013	0.338							Model 1
%CER18:1	0 [-0.03 - 0.02]	0.826	0.956	0.093							Model 1
%CER20:1	0.03 [0.01 - 0.06]	0.009	0.058	0.252							Model 1
%CER20:1	0 [-0.03 - 0.03]	0.944	0.956	0.847							Model 1
%CER22:0	0.02 [0 - 0.05]	0.077	0.2	0.081							Model 1
%CER22:1	0 [-0.02 - 0.03]	0.735	0.956	0.581							Model 1
%CER24:0	-0.02 [-0.05 - 0]	0.066	0.2	0.15							Model 1
%CER24:1	0.01 [-0.02 - 0.04]	0.443	0.902	0.194							Model 1
%CER26:0	-0.01 [-0.04 - 0.02]	0.555	0.902	0.268							Model 1
%CER26:1	-0.01 [-0.03 - 0.02]	0.53	0.902	0.476							Model 1
Total %CER	0 [-0.03 - 0.03]	0.956	0.956	0.037							Model 1
%CER14:0	-0.02 [-0.04 - 0.01]	0.184	0.886	0.058							Model 2
%CER16:0	0.01 [-0.02 - 0.04]	0.467	0.886	0.076							Model 2
%CER18:0	0.02 [-0.01 - 0.05]	0.19	0.886	0.133							Model 2
%CER18:1	0 [-0.03 - 0.03]	0.886	0.886	0.039							Model 2
%CER20:1	0.02 [-0.01 - 0.04]	0.219	0.886	0.182							Model 2
%CER20:1	0 [-0.03 - 0.04]	0.829	0.886	0.881							Model 2
%CER22:0	0 [-0.02 - 0.03]	0.75	0.886	0.099							Model 2
%CER22:1	-0.01 [-0.03 - 0.02]	0.679	0.886	0.589							Model 2
%CER24:0	-0.01 [-0.04 - 0.02]	0.448	0.886	0.184							Model 2
%CER24:1	0 [-0.02 - 0.03]	0.792	0.886	0.297							Model 2
%CER26:0	0 [-0.04 - 0.02]	0.735	0.886	0.451							Model 2
%CER26:1	-0.01 [-0.04 - 0.02]	0.541	0.886	0.285							Model 2
Total %CER	0 [-0.03 - 0.02]	0.781	0.886	0.136							Model 2
CER16:0/24:0	0.01 [-0.01 - 0.04]	0.41	0.452	0.285							Model 1
CER18:0/24:0	0.04 [0.02 - 0.07]	0.001	0.003	0.306							Model 1
CER20:0/24:0	0.04 [0.01 - 0.06]	0.005	0.01	0.231							Model 1
CER24:1/24:0	0.02 [-0.01 - 0.04]	0.208	0.312	0.197							Model 1
CER18:0/16:0	0.05 [0.02 - 0.07]	<0.001	<0.001	0.015	0.06 [0.03 - 0.1]	<0.001	<0.001	0.03 [-0.01 - 0.06]	0.183	0.22	Model 1

CER22:0/16:0	0.01 [-0.02 - 0.04]	0.452	0.452	0.019	0.04 [0 - 0.07]	0.038	0.058	-0.03 [-0.07 - 0.01]	0.132	0.198	Model 1
CER16:0/24:0	0.01 [-0.02 - 0.04]	0.464	0.65	0.398							Model 2
CER18:0/24:0	0.02 [-0.01 - 0.05]	0.215	0.512	0.137							Model 2
CER20:0/24:0	0.02 [-0.01 - 0.05]	0.192	0.512	0.162							Model 2
CER24:1/24:0	0.01 [-0.02 - 0.03]	0.65	0.65	0.259							Model 2
CER18:0/16:0	0.02 [-0.01 - 0.05]	0.256	0.512	0.007	0.03 [-0.01 - 0.07]	0.115	0.483	-0.01 [-0.06 - 0.03]	0.635	0.805	Model 2
CER22:0/16:0	-0.01 [-0.03 - 0.02]	0.598	0.65	0.042	0.01 [-0.03 - 0.05]	0.539	0.683	-0.04 [-0.08 - 0]	0.078	0.468	Model 2
CER score	0.01 [0 - 0.02]	0.002	0.002	<0.001	0.02 [0.01 - 0.03]	<0.001	<0.001	0 [-0.01 - 0.01]	0.902	0.902	Model 1
CER score	0.01 [0 - 0.02]	0.124	0.124	<0.001	0.01 [0 - 0.03]	0.058	0.058	0 [-0.01 - 0.02]	0.932	0.932	Model 2

Supplementary Table 3: Cardiometabolic disease indicators as mediators between absolute and relative concentrations of CERs, CER ratios and the CER score, and WMH load

Exposure	Effect	CVD	Diabetes	Hypertension
CER14:0	Direct	0.003 [-0.022 - 0.028]	0.004 [-0.020 - 0.030]	0.003 [-0.023 - 0.028]
	Indirect	-0.001 [-0.002 – 0.000]	-0.002 [-0.005 - -0.001]	-0.002 [-0.006 - 0.001]
	Percentage mediated	-0.74%	-6.90%	1.34%
CER16:0	Direct	0.033 [0.007 - 0.059]	0.034 [0.006 - 0.060]	0.030 [0.005 - 0.056]
	Indirect	-0.001 [-0.003 - 0.000]	-0.001 [-0.003 - 0.000]	0.000 [-0.003 - 0.003]
	Percentage mediated	-3.52%	-3.33%	-0.36%
CER18:0	Direct	0.056 [0.029 - 0.082]	0.054 [0.028 - 0.08]	0.046 [0.021 - 0.072]
	Indirect	-0.001 [-0.002 – 0.000]	0.002 [0.000 - 0.005]	0.010 [0.006 - 0.015]
	Percentage mediated	-1.03%	4.16%	17.74%
CER18:1	Direct	0.014 [-0.011 - 0.038]	0.015 [-0.012 - 0.041]	0.016 [-0.008 - 0.041]
	Indirect	0.000 [-0.001 - 0.001]	0.000 [-0.002 - 0.001]	-0.002 [-0.006 - 0.001]
	Percentage mediated	-0.94%	-1.81%	-9.71%
CER20:0	Direct	0.046 [0.020 - 0.072]	0.044 [0.017 - 0.070]	0.039 [0.013 - 0.064]
	Indirect	-0.001 [-0.003 - 0.000]	0.001 [0.000 - 0.003]	0.006 [0.003 - 0.010]
	Percentage mediated	-2.01%	2.94%	13.02%
CER20:1	Direct	0.017 [-0.014 - 0.047]	0.019 [-0.010 - 0.049]	0.012 [-0.018 - 0.042]
	Indirect	-0.001 [-0.003 - 0.000]	-0.001 [-0.004 - 0.000]	0.003 [-0.001 - 0.008]
	Percentage mediated	-2.54%	-4.96%	13.14%
CER22:0	Direct	0.032 [0.005 - 0.058]	0.028 [0.005 - 0.053]	0.024 [-0.001 - 0.050]
	Indirect	-0.001 [-0.003 - 0.000]	0.001 [0.000 - 0.003]	0.005 [0.002 - 0.009]
	Percentage mediated	-3.19%	3.04%	16.89%
CER22:1	Direct	0.028 [0.001 - 0.053]	0.029 [0.004 - 0.054]	0.02 [-0.008 - 0.046]
	Indirect	-0.001 [-0.003 - 0.000]	-0.001 [-0.002 - 0.001]	0.003 [0.000 - 0.007]
	Percentage mediated	-3.88%	-1.85%	13.63%

	Direct	0.014 [-0.013 - 0.041]	0.013 [-0.015 - 0.039]	0.011 [-0.012 - 0.037]
CER24:0	Indirect	-0.001 [-0.003 - 0.000]	-0.001 [-0.003 - 0.000]	0.001 [-0.002 - 0.004]
	Percentage mediated	-4.98%	-5.23%	4.29%
	Direct	0.031 [0.005 - 0.058]	0.030 [0.004 - 0.055]	0.024 [-0.002 - 0.050]
CER24:1	Indirect	-0.001 [-0.003 - 0.000]	0.000 [-0.002 - 0.001]	0.006 [0.002 - 0.009]
	Percentage mediated	-4.10%	-1.52%	19.28%
	Direct	0.001 [-0.025 - 0.031]	0.001 [-0.029 - 0.028]	0.002 [-0.027 - 0.028]
CER26:0	Indirect	0.000 [-0.002 - 0.001]	-0.001 [-0.003 - 0.001]	0.000 [-0.004 - 0.003]
	Percentage mediated	0.01%	0.13%	1.53%
	Direct	0.013 [-0.011 - 0.039]	0.015 [-0.012 - 0.044]	0.012 [-0.017 - 0.039]
CER26:1	Indirect	-0.001 [-0.003 - 0.000]	-0.003 [-0.006 - -0.001]	0.000 [-0.003 - 0.004]
	Percentage mediated	4.54%	-13.86%	1.57%
	Direct	0.027 [0.000 - 0.054]	0.025 [-0.002 - 0.050]	0.021 [-0.006 - 0.049]
Total CER	Indirect	-0.001 [-0.003 - 0.000]	-0.001 [-0.002 - 0.001]	0.003 [0.000 - 0.007]
	Percentage mediated	-4.68%	-1.84%	13.07%
	Direct	-0.022 [-0.047 - 0.004]	-0.021 [-0.045 - 0.006]	-0.019 [-0.043 - 0.007]
%CER14:0	Indirect	0.000 [-0.001 - 0.001]	-0.002 [-0.004 - 0]	-0.005 [-0.009 - -0.002]
	Percentage mediated	-0.08%	6.69%	20.00%
	Direct	0.001 [-0.024 - 0.024]	0.003 [-0.023 - 0.028]	0.006 [-0.018 - 0.032]
%CER16:0	Indirect	0.000 [-0.001 - 0.001]	-0.001 [-0.003 – 0.000]	-0.005 [-0.009 - -0.002]
	Percentage mediated	0.12%	-1.47%	-13.33%
	Direct	0.044 [0.019 - 0.069]	0.041 [0.015 - 0.067]	0.034 [0.009 - 0.058]
%CER18:0	Indirect	0.000 [-0.001 - 0.001]	0.004 [0.001 - 0.007]	0.010 [0.006 - 0.015]
	Percentage mediated	0.49%	7.65%	23.37%
	Direct	-0.003 [-0.028 - 0.021]	-0.003 [-0.028 - 0.024]	-0.001 [-0.026 - 0.023]
%CER18:1	Indirect	0.000 [-0.001 - 0.001]	0.000 [-0.001 - 0.001]	-0.003 [-0.007 - 0.000]
	Percentage mediated	-0.10%	0.16%	14.48%
	Direct	0.033 [0.009 - 0.059]	0.030 [0.004 - 0.055]	0.028 [0.003 - 0.053]

%CER20:0	Indirect	0.000 [-0.001 - 0.001]	0.003 [0.001 - 0.005]	0.005 [0.003 - 0.009]
	Percentage mediated	0.10%	8.42%	16.83%
	Direct	0.000 [-0.029 - 0.029]	0.000 [-0.030 - 0.030]	-0.002 [-0.032 - 0.027]
%CER20:1	Indirect	0.000 [-0.002 - 0.001]	0.000 [-0.002 - 0.002]	0.001 [-0.003 - 0.005]
	Percentage mediated	0.08%	0.26%	0.83%
	Direct	0.022 [-0.002 - 0.044]	0.019 [-0.006 - 0.044]	0.016 [-0.009 - 0.041]
%CER22:0	Indirect	0.000 [-0.001 - 0.001]	0.004 [0.001 - 0.007]	0.007 [0.003 - 0.010]
	Percentage mediated	0.61%	15.41%	28.16%
	Direct	0.005 [-0.02 - 0.028]	0.007 [-0.020 - 0.031]	0.001 [-0.026 - 0.027]
%CER22:1	Indirect	0.000 [-0.001 - 0.001]	0.000 [-0.002 - 0.001]	0.001 [-0.002 - 0.004]
	Percentage mediated	-0.18%	-0.97%	3.73%
	Direct	-0.024 [-0.049 - 0.001]	-0.022 [-0.046 - 0.002]	-0.017 [-0.041 - 0.008]
%CER24:0	Indirect	0.000 [-0.001 - 0.001]	-0.002 [-0.004 - 0]	-0.007 [-0.010 - -0.003]
	Percentage mediated	0.10%	7.48%	26.59%
	Direct	0.010 [-0.015 - 0.037]	0.009 [-0.016 - 0.034]	0.005 [-0.019 - 0.030]
%CER24:1	Indirect	0.000 [-0.001 - 0.001]	0.000 [-0.001 - 0.002]	0.005 [0.002 - 0.009]
	Percentage mediated	-0.11%	0.55%	27.61%
	Direct	-0.008 [-0.039 - 0.021]	-0.010 [-0.039 - 0.020]	-0.006 [-0.035 - 0.023]
%CER26:0	Indirect	0.000 [-0.001 - 0.002]	0.000 [-0.002 - 0.003]	-0.001 [-0.005 - 0.002]
	Percentage mediated	-0.55%	-0.49%	5.15%
	Direct	-0.008 [-0.034 - 0.018]	-0.006 [-0.033 - 0.021]	-0.007 [-0.035 - 0.020]
%CER26:1	Indirect	0.000 [-0.001 - 0.001]	-0.002 [-0.005 – 0.000]	-0.002 [-0.006 - 0.001]
	Percentage mediated	0.42%	12.45%	11.58%
	Direct	-0.001 [-0.025 - 0.026]	-0.002 [-0.026 - 0.023]	-0.001 [-0.026 - 0.024]
Total %CER	Indirect	0.000 [-0.001 - 0.001]	0.000 [-0.001 - 0.002]	0.000 [-0.003 - 0.003]
	Percentage mediated	-0.01%	-0.45%	1.46%
	Direct	0.046 [0.021 - 0.072]	0.043 [0.018 - 0.07]	0.033 [0.008 - 0.058]
CER18:0/16:0	Indirect	0 [0.000 - 0.002]	0.004 [0.001 - 0.008]	0.014 [0.009 - 0.02]

	Percentage mediated	0.55%	8.22%	29.98%
	Direct	0.009 [-0.018 - 0.035]	0.006 [-0.019 - 0.030]	0.002 [-0.026 - 0.028]
CER22:0/16:0	Indirect	0.000 [-0.001 - 0.001]	0.003 [0.001 - 0.005]	0.008 [0.005 - 0.012]
	Percentage mediated	0.77%	15.12%	43.34%
	Direct	0.011 [-0.014 - 0.036]	0.011 [-0.015 - 0.035]	0.011 [-0.014 - 0.036]
CER16:0/24:0	Indirect	0.000 [-0.001 - 0.001]	0.000 [-0.001 - 0.002]	-0.001 [-0.004 - 0.002]
	Percentage mediated	0.13%	0.95%	-1.95%
	Direct	0.029 [0.003 - 0.055]	0.040 [0.014 - 0.065]	0.033 [0.006 - 0.060]
CER18:0/24:0	Indirect	0.013 [0.005 - 0.021]	0.003 [0.001 - 0.007]	0.010 [0.006 - 0.015]
	Percentage mediated	30.58%	8.71%	24.16%
	Direct	0.035 [0.010 - 0.060]	0.033 [0.007 - 0.058]	0.028 [0.004 - 0.054]
CER20:0/24:0	Indirect	0.000 [-0.001 - 0.001]	0.003 [0.001 - 0.006]	0.007 [0.003 - 0.011]
	Percentage mediated	0.25%	8.71%	18.84%
	Direct	0.016 [-0.010 - 0.041]	0.014 [-0.011 - 0.041]	0.010 [-0.015 - 0.034]
CER24:1/24:0	Indirect	0.000 [-0.001 - 0.001]	0.001 [0.000 - 0.003]	0.006 [0.003 - 0.010]
	Percentage mediated	0.00%	4.34%	31.61%
	Direct	0.014 [0.005 - 0.022]	0.013 [0.004 - 0.022]	0.011 [0.003 - 0.019]
CER score	Indirect	0.000 [-0.001 - 0.000]	0.000 [0.000 - 0.001]	0.002 [0.001 - 0.004]
	Percentage mediated	-2,43%	2.99%	17.35%
